# Physical activity and IgG N-glycosylation in medical students: a cross-sectional study

**DOI:** 10.3325/cmj.2026.67.156

**Published:** 2026-06

**Authors:** Stipe Vidović, Ivanka Maduna, Matko Fančović, Nina Šimunić-Briški, Maja Hanić, Petar Šušnjara, Gordan Lauc, Marija Heffer, Lada Zibar

**Affiliations:** 1 Faculty of Medicine, Josip Juraj Strossmayer University of Osijek, Osijek, Croatia; 2 Genos Glycoscience Research Laboratory, Zagreb, Croatia; 3 Department of Interdisciplinary Sciences, Faculty of Kinesiology, Josip Juraj Strossmayer University of Osijek, Osijek, Croatia; 4 Department of Medical Biology, Faculty of Medicine, Josip Juraj Strossmayer University of Osijek, Osijek, Croatia; 5 Department of Pathophysiology, Faculty of Medicine, Josip Juraj Strossmayer University of Osijek, Osijek, Croatia; 6 Clinic for Internal Medicine, University Hospital Merkur, Zagreb, Croatia

## Abstract

**Aim:**

To examine differences in IgG N-glycan composition between physical activity (PA) categories and to assess associations between PA domains and IgG N-glycan traits in medical students.

**Methods:**

This cross-sectional study enrolled first-year and second-year medical students at the University Josip Juraj Strossmayer, Osijek. PA was assessed using the International Physical Activity Questionnaire-Short Form (IPAQ-SF), capturing walking, moderate, and vigorous PA, total PA, and sitting time. Participants were classified as health-enhancing physical activity (HEPA)-active or non-HEPA-active. IgG was isolated from plasma, and N-glycan composition was assessed using capillary gel electrophoresis with laser-induced fluorescence detection. Twenty-seven glycan peaks and eight derived glycan traits (G0, G1, G2, S0, S1, S2, B, and CF) were quantified.

**Results:**

Seventy-nine students (56 women; median age, 20 [range, 18-25] years) were enrolled: 23 were HEPA-active and 56 non-HEPA-active. The groups did not significantly differ in individual IgG N-glycan peaks or derived glycan traits. In unadjusted analyses, vigorous PA was weakly positively correlated with G1 (ρ = 0.24, *P* = 0.034) and total PA with G1 (ρ = 0.27, *P* = 0.017), while total PA was negatively correlated with G2 (ρ = −0.34, *P* = 0.002). However, none of these associations remained significant after adjustment for multiple testing.

**Conclusion:**

Self-reported PA was not associated with IgG N-glycosylation after correction, and no differences were observed between PA categories. These findings indicate no robust association between PA and the IgG glycome.

Physical activity (PA) is a well-established modulator of immune function and systemic inflammation. It is associated with a reduced risk of cardiovascular, autoimmune, and inflammatory diseases (1,2). Beyond its effects on classical inflammatory biomarkers, increasing attention has been directed toward the influence of PA on post-translational modifications of immune effector molecules (1,3,4). In this context, immunoglobulin G (IgG) N-glycosylation has emerged as a sensitive and integrative biomarker of immune status, reflecting the balance between pro- and anti-inflammatory immune signaling (5,6).

IgG N-glycans are structurally complex, predominantly biantennary complex-type oligosaccharides covalently attached to the conserved asparagine 297 residue within the Fc region of the IgG heavy chain. These glycans share a common N-glycan core structure but exhibit considerable microheterogeneity through the presence or absence of terminal galactose and sialic acid residues, as well as core fucosylation and bisecting N-acetylglucosamine (5-7). This structural variability critically influences the three-dimensional conformation of the Fc domain and thereby modulates antibody effector functions, including Fc receptor binding affinity and complement activation (5,7). Consequently, specific glycosylation patterns have been linked to distinct functional and immunological outcomes (7). Decreased galactosylation and sialylation or increased agalactosylation have been associated with chronic inflammation, aging, and cardiometabolic disease, whereas higher levels of galactosylation and sialylation are markers of a more anti-inflammatory IgG phenotype (8-11). Additional structural features, including core fucosylation and bisecting N-acetylglucosamine, further contribute to the functional diversity of the IgG glycome (12).

Because IgG N-glycosylation is closely linked to systemic inflammatory and metabolic states, it represents a plausible molecular interface through which habitual lifestyle behaviors may be reflected in immune function (12-14). Regular physical activity exerts broad immunometabolic effects, including reductions in low-grade inflammation, improved metabolic regulation, and modulation of innate and adaptive immune responses (1,2). In general, more anti-inflammatory physiological states have been associated with lower IgG agalactosylation and higher galactosylation and sialylation, whereas pro-inflammatory states such as obesity, aging, and chronic disease tend to show the opposite pattern (8-11). These observations support the biological premise that habitual PA may be associated with variation in the IgG glycome, although such effects are likely to be indirect and cumulative.

Despite this biological plausibility, the relationship between physical activity and IgG N-glycosylation remains insufficiently characterized (15). Existing evidence is limited and heterogeneous, with most studies focusing on clinical populations, older adults, athletes, or structured exercise interventions rather than routine physical activity patterns in young, apparently healthy individuals (16-19). Consequently, it remains unclear whether differences in everyday PA are already reflected in IgG glycosylation at an early stage of adult life, before overt metabolic or inflammatory diseases become apparent.

Young adulthood may be a particularly informative period in which to investigate this question, as long-term health behaviors are being established while subclinical biological variation may still be detectable in the absence of manifest disease (20,21). Medical students represent a relevant subgroup in this context: despite relatively high health literacy, they are frequently exposed to sedentary academic routines and may not consistently achieve recommended levels of PA (22-24). Therefore, the aim of this cross-sectional study was to investigate the association between PA and IgG N-glycosylation patterns in young adults enrolled in medical training. We hypothesized that higher levels of PA would be associated with a shift toward a less pro-inflammatory glycosylation profile, characterized by lower agalactosylation and higher galactosylation and sialylation. Other glycan features, including core fucosylation and bisecting N-acetylglucosamine, were analyzed in an exploratory manner without predefined directional hypotheses.

## METHODS

### Participants and recruitment

This cross-sectional study enrolled first- and second-year medical students at the Faculty of Medicine, University Josip Juraj Strossmayer (JJS), Osijek, Croatia. At this institution, students in the first two years of the medical program attend a mandatory PA course as part of the official curriculum. This course comprises structured kinesiology-based PA, providing a relatively homogeneous and well-defined population for the assessment of PA patterns.

Participants were recruited using a convenience sampling. All eligible students attending their scheduled teaching activities on a regular academic day (December 22, 2023) were invited to participate in the study. Prior to enrollment, students were informed about the study objectives and procedures, both verbally and in written form. Written informed consent was obtained from all participants before any data collection.

Participation in the study was entirely voluntary, and no incentives were provided. To ensure confidentiality and anonymity, each participant was assigned a unique, randomly generated identification code, which was used for all questionnaires and biological samples. No personally identifiable information was accessible to the researchers during data analysis. No other inclusion criteria were required. The study protocol was approved by the Institutional Ethics Committee of the Faculty of Medicine, University JJS of Osijek (2158-61-46-23-147).

### Assessment of physical activity

PA was assessed using the International Physical Activity Questionnaire – Short Form (IPAQ-SF) (25,26). The IPAQ-SF is a validated and internationally standardized self-report instrument designed to assess the frequency (days per week) and duration (minutes per day) of PA performed during the previous seven days. The questionnaire captures time spent in vigorous-intensity activity, moderate-intensity activity, and walking, while sitting time is recorded separately and not included in the total PA score. Only activity bouts lasting at least ten minutes were considered. Data were processed according to the official IPAQ scoring protocol, including truncation of activity duration values exceeding 180 minutes per day. PA was expressed as metabolic equivalent minutes per week (MET-min/week) by multiplying activity duration, frequency, and standardized MET values (vigorous activity: 8.0 METs; moderate activity: 4.0 METs; walking: 3.3 METs). Total PA was calculated as the sum of all activity domains. According to IPAQ-SF classification criteria, participants were categorized into health-enhancing physical activity (HEPA)-active and non-HEPA-active groups. Individuals were classified as HEPA-active if they met at least one of the following thresholds: (i) vigorous-intensity PA on ≥3 days per week accumulating ≥1500 MET-min/week or (ii)≥7 days of any combination of walking, moderate- or vigorous-intensity activity achieving ≥3000 MET-min/week. Participants who did not meet these criteria were classified as non-HEPA active. The validated Croatian version of the IPAQ-SF was used (27).

### IgG N-glycan assessment

After questionnaire completion, venous blood samples were collected into EDTA tubes. Plasma was separated by centrifugation and stored at −80 °C until analysis. IgG N-glycans were assessed using capillary gel electrophoresis with laser-induced fluorescence detection (CGE-LIF), following established analytical protocols with minor modifications (28,29). The structural annotation and nomenclature of individual IgG N-glycan peaks are summarized in Supplemental Table 1(Supplementary Table 1). A representative electropherogram illustrating the separation of IgG N-glycans is shown in Supplemental Figure 1(Supplementary Figure 1).

IgG was isolated from plasma samples using a Protein G monolithic plate. N-glycans were enzymatically released using N-glycosidase F (PNGase F; Promega, Madison, WI, USA) after prior glycoprotein denaturation. Released glycans were fluorescently labeled with 8-aminopyrene-1,3,6-trisulfonic acid (APTS; Synchem, Felsberg, Germany) by reductive amination. Excess label, residual proteins, and salts were removed by hydrophilic interaction liquid chromatography-based solid phase extraction. Glycans were then separated and profiled an ABI3500 Genetic Analyzer (Applied Biosystems, Waltham, MA, USA) with a 50-cm, 8-capillary array filled with POP-7 polymer. Electropherograms were manually integrated using Empower 3 software (Waters, Milford, MA, USA), which enabled detection and relative quantification of 27 distinct IgG N-glycan peaks. The numerical values of all quantified IgG N-glycan peaks (P1-P27) for all study participants are shown in Supplemental Table 2(Supplementary Table 2).

Derived glycan traits were calculated to reflect shared structural and functional features and expressed as relative abundances (%) within the total IgG glycome. Galactosylation traits included agalactosylation (G0), monogalactosylation (G1), and digalactosylation (G2). Sialylation traits included asialylation (S0), monosialylation (S1), and disialylation (S2). Additional structural traits included bisecting N-acetylglucosamine (B) and core fucosylation (CF). The specific glycan peaks included in each derived trait are shown in Supplemental Table 3(Supplementary Table 3).

### Statistical analysis

Numerical variables are presented as medians with interquartile ranges (IQR). Normality of distribution was assessed using the Shapiro-Wilk test. Between-group differences in individual IgG N-glycan peaks and derived glycan traits were evaluated using the Mann-Whitney U test, with effect sizes expressed as rank-biserial correlation coefficients (r). Correlations between IgG N-glycan measures and IPAQ-derived PA variables were assessed using Spearman’s rank correlation coefficient (ρ). To account for potential confounding by body mass index (BMI), BMI-adjusted associations were examined using partial Spearman correlations, implemented via residuals obtained by regressing glycan traits and PA variables on BMI. Potential effect modification by BMI was examined using linear regression models including total PA, BMI category, and their interaction term. *P* values were adjusted using the Benjamini-Hochberg false discovery rate (FDR) procedure separately within each family of tests. Statistical significance was defined as FDR-adjusted *P* < 0.05. Statistical analysis was performed using jamovi (version 2.6; The jamovi project) and R (version 4.4.0; R Core Team).

## RESULTS

Initially, 82 participants were enrolled, but three were excluded due to incomplete IPAQ-SF questionnaires. Consequently, the final analysis included 79 participants (56 women). The median age was 20 (range, 18-25) years. Forty-three participants were first-year medical students, while 36 were in their second year. Based on BMI, 68.4% of participants were classified as having normal weight (18.5-24.9 kg/m^2^), 12.7% were underweight (<18.5 kg/m^2^), 12.7% were overweight (25.0-29.9 kg/m^2^), and 5.1% were obese (≥30 kg/m^2^).

According to the IPAQ-SF classification, 23 participants were categorized as HEPA-active and 56 as non-HEPA-active. Participants’ IPAQ-SF scores and PA categories are presented in Table 1.

**Table 1:** Participants’ International Physical Activity Questionnaire – Short Form scores and physical activity categories (N = 79)

Characteristic	Median (IQR) or n (%)
PA (MET min/week)	
walking	693 (462-1270.5)
moderate	320 (120-620)
vigorous	480 (200-1440)
Total MET min/week	1773 (1334-3306)
Sitting time (hours)	
<4	2 (2.5)
4-6	12 (15.2)
6-8	21 (26.6)
8-10	21 (26.6)
>10	23 (29.1)
IPAQ PA category	
HEPA-active	23 (29.1)
Non-HEPA active	56 (70.9)

*Abbreviations: MET – metabolic equivalent; HEPA – health-enhancing physical activity; IQR – interquartile range.

HEPA-active and non-HEPA-active participants did not significantly differ in the relative abundances of 27 individual IgG N-glycan peaks (Table 2). After adjustment for multiple testing using the Benjamini-Hochberg FDR procedure, the two groups significantly differed in none of the individual IgG N-glycan peaks (P1-P27) (all FDR-adjusted *P*-values = 0.893).

**Table 2:** The relative abundances of individual IgG N-glycan peaks (P1-P27) in HEPA-active and non-HEPA-active participants*

	HEPA active (n = 23)^‡^	Non-HEPA active (n = 56)			
	median (IQR)	median (IQR)	U	*P*	r†
P1	0.37 (0.35-0.44)	0.41 (0.36-0.45)	543	0.278	−0.16
P2	0.21 (0.19-0.25)	0.22 (0.19-0.25)	621	0.808	−0.04
P3	2.23 (1.82-2.57)	2.14 (1.92-2.56)	629	0.876	−0.02
P4	1.83 (1.60-2.01)	1.76 (1.59-2.05)	670	0.783	0.04
P5	0.16 (0.14-0.17)	0.16 (0.15-0.18)	568	0.415	−0.12
P6	0.03 (0.02-0.05)	0.04 (0.03-0.04)	585	0.528	−0.09
P7	0.27 (0.26-0.28)	0.26 (0.24-0.29)	747	0.269	0.16
P8	2.22 (1.99-2.46)	2.22 (1.94-2.40)	686	0.654	0.07
P9	0.26 (0.24-0.29)	0.26 (0.24-0.29)	657	0.893	0.02
P10	0.55 (0.49-0.62)	0.56 (0.50-0.63)	584	0.521	−0.09
P11	0.28 (0.26-0.30)	0.30 (0.26-0.32)	547	0.298	−0.15
P12	11.29 (10.32-13.24)	11.92 (10.49-13.42)	610	0.718	−0.05
P13	2.37 (2.12-2.66)	2.37 (2.09-2.51)	686	0.654	0.07
P14	0.31 (0.29-0.34)	0.29 (0.27-0.32)	800	0.093	0.24
P15	13.87 (13.00-16.82)	14.23 (11.59-16.33)	689	0.631	0.07
P16	0.40 (0.24-0.61)	0.41 (0.30-0.55)	586	0.535	−0.09
P17	0.25 (0.17-0.34)	0.26 (0.21-0.31)	611	0.726	−0.05
P18	2.09 (1.79-2.45)	2.05 (1.85-2.38)	626	0.850	−0.03
P19	0.22 (0.18-0.25)	0.22 (0.20-0.25)	580	0.493	−0.10
P20	0.28 (0.25-0.31)	0.26 (0.23-0.31)	784	0.132	0.22
P21	19.53 (18.19-20.63)	18.75 (17.65-19.86)	780	0.144	0.21
P22	11.50 (10.90-12.30)	11.13 (10.18-11.99)	730	0.356	0.13
P23	4.91 (4.17-5.11)	4.90 (4.54-5.28)	601	0.646	−0.07
P24	0.57 (0.52-0.62)	0.55 (0.52-0.60)	707	0.500	0.10
P25	0.14 (0.13-0.16)	0.14 (0.13-0.17)	615	0.758	−0.05
P26	21.19 (19.82-22.81)	22.00 (19.48-24.35)	548	0.303	−0.15
P27	1.56 (1.40-1.72)	1.59 (1.47-1.84)	580	0.493	−0.10

*Abbreviations: HEPA – health-enhancing physical activity; IQR – interquartile range; U – Mann-Whitney U test statistic; r – rank-biserial correlation coefficient (effect size).

†Positive rank-biserial correlation values indicate higher ranks in the HEPA-active group.

‡HEPA active participants are those who, according to IPAQ-SF, met at least one of the following criteria: engaging in vigorous-intensity physical activity on ≥3 days per week accumulating ≥1500 MET-min/week, or participating in ≥7 days of any combination of walking, moderate-, or vigorous-intensity activities achieving ≥3000 MET-min/week. Participants not meeting these thresholds were classified as non-HEPA active.

HEPA-active and non-HEPA-active participants did not significantly differ in any derived IgG N-glycan trait (G0, G1, G2, S0, S1, S2, B, and CF) (Table 3). After adjustment for multiple testing across eight derived IgG glycan traits, the groups did not differ in any of the traits, with FDR-adjusted *P*-values ranging from 0.872 to 0.996.

**Table 3:** Derived IgG N-glycan traits in HEPA-active and non-HEPA-active participants*

	HEPA-active (n = 23)	Non-HEPA active (n = 56)			
	median (IQR)	median (IQR)	U	*P*	r†
G0	16.46 (14.92-19.32)	16.44 (14.00-19.43)	675	0.742	0.05
G1	37.28 (36.21-39.24)	36.82 (35.15-38.11)	793	0.109	0.23
G2	22.97 (21.47-24.57)	23.68 (21.17-26.13)	550	0.313	−0.15
S0	22.47 (20.92-23.97)	23.02 (20.74-24.85)	632	0.901	−0.02
S1	17.71 (16.43-19.38)	17.98 (16.50-19.98)	621	0.808	−0.04
S2	4.50 (4.04-5.40)	4.64 (4.08-5.20)	630	0.884	−0.02
B	14.99 (14.12-16.25)	14.93 (13.85-16.08)	645	0.996	<0.01
CF	96.59 (96.08-96.81)	96.32 (96.11-96.74)	692	0.608	0.07

*Abbreviations: HEPA – health-enhancing physical activity; IQR – interquartile range; U – Mann-Whitney U test statistic; r – rank-biserial correlation coefficient (effect size).

†Positive rank-biserial correlation values indicate higher ranks in the HEPA-active group.

Spearman correlation analyses between individual IgG N-glycan peaks (P1-P27) and PA domains derived from the IPAQ questionnaire revealed several significant correlations (*P* < 0.05) (Supplemental Table 4(Supplementary Table 4)). After adjustment for multiple testing, none of the associations remained significant.

We also assessed the correlations between derived IgG N-glycan traits and PA domains. In unadjusted analyses, nominal associations included a weak positive correlation between vigorous PA and G1 (ρ = 0.24, *P* = 0.034), a weak positive correlation between total PA and G1 (ρ = 0.27, *P* = 0.017), and a moderate negative correlation between total PA and G2 (ρ = −0.34, *P* = 0.002). No other associations were observed (Supplemental Table 5(Supplementary Table 5)).

After BMI adjustment, similar nominal associations were identified for total PA with G1 and G2, and for walking with G2; however, after FDR correction none remained significant (all adjusted *P* ≥ 0.218). A nominal association was also observed between walking and P22. Interaction analyses suggested potential PA-BMI effects for G1, G2, and S1, but these did not persist after multiple testing correction (all adjusted *P* ≥ 0.262).

## DISCUSSION

In this study, self-reported PA was not associated with individual IgG N-glycan peaks or derived glycan traits after correction for multiple testing. Although several nominal associations were observed, none remained significant after FDR adjustment. Therefore, our findings do not support the hypothesis that higher levels of PA would be associated with a shift in IgG glycosylation toward a less pro-inflammatory profile.

These findings can be explained by the temporal mismatch between the exposure and outcome measures. The IPAQ-SF reflects self-reported PA over the previous seven days, whereas IgG glycosylation likely captures longer-term immune and metabolic regulation (15,27). In young and healthy individuals, short-term variation in activity may therefore be insufficient to produce detectable differences in the IgG glycome. In addition, the relative homogeneity of the study population with respect to age, educational setting, and overall health status may have further reduced variability in both PA and glycan traits, thereby limiting statistical power to detect modest associations.

Although nominal associations suggested that vigorous and total PA were positively related to monogalactosylated glycans and inversely related to digalactosylated glycans, these findings should be interpreted cautiously, as none remained statistically significant after FDR correction. Moreover, no consistent associations were observed for other glycan features, including sialylation, bisecting GlcNAc, and core fucosylation, suggesting that any relationship between PA and the IgG glycome was not generalized across multiple structural domains.

Direct comparison with previous studies is complicated by substantial differences in study design, participant characteristics, baseline health status, and the type and duration of PA exposure. Previous intervention studies reported heterogeneous findings, underscoring the context-dependent nature of this relationship. In a prospective study, first-year male students of kinesiology completed six weeks of repeated-sprint training (intense PA). At the end of the intervention, IgG N-glycan profiles showed proinflammatory trends. However, after an additional six weeks of recovery period, glycan profiles shifted to an anti-inflammatory profile, characterized by decreased agalactosylation and increased digalactosylation and sialylation (18). Similar trends were observed in a young female cohort (aged 25 on average), who engaged in intense PA while reducing caloric intake. At the end of the intervention, a proinflammatory IgG profile was observed, returning to the baseline after the same length of recovery period (19). In contrast, a three-month intervention in previously inactive, mid-aged overweight individuals demonstrated increased monogalactosylated and decreased digalactosylated glycans, alongside changes in other glycan traits suggestive of a less anti-inflammatory IgG glycome, without a recovery period, aligning more closely with the nominal trends observed in our cohort (16). Additionally, a cross-sectional study examining physical activity of varying intensity reported higher levels of agalactosylated IgG structures (FA2) and lower levels of digalactosylated structures (FA2G2, FA2BG2) in intensively-training and competing athletes compared with moderately active, non-competing individuals (30). Accordingly, the effects of PA on IgG glycosylation are complex and likely modulated by exercise intensity, duration, and baseline metabolic status. From a biological perspective, IgG glycosylation reflects long-term regulation of immune and inflammatory processes and is strongly influenced by aging and systemic inflammation (15-19,30,31). Although PA modulates inflammatory pathways, its effects on IgG glycosylation are likely indirect and cumulative (15-19,30,31). This may partly explain the absence of detectable associations in the present cohort.

BMI-adjusted analyses yielded comparable results, with nominal associations for G1 and G2, which again did not withstand multiple-testing correction. No significant interaction between PA and BMI category was observed, suggesting that BMI did not meaningfully modify the association between PA and IgG glycosylation. Given the established relationship between adiposity and a more pro-inflammatory IgG glycome, this lack of interaction is likely explained by the relatively narrow distribution of BMI and PA levels in the sample rather than by the absence of a broader biological effect (8,12,32).

Results interpretation needs to take into account the characteristics of the study population. Participants were young adults recruited from a relatively homogeneous university setting. IgG glycosylation is known to vary with age and to reflect cumulative influences of biological aging, metabolic health, inflammatory status, hormonal factors, and environmental exposures (8,33). Younger individuals generally have more stable glycan profiles with less interindividual variability than older populations (32). This may have reduced the study’s ability to detect PA-related differences. In addition, the IPAQ-SF captures recent self-reported activity and may be less informative for long-term immunometabolic regulation than objective measures, long-term training history, or cardiorespiratory fitness (8,32,33).

Several limitations should be acknowledged. First, the cross-sectional design precludes causal inference between PA and plasma-derived IgG N-glycosylation. Future longitudinal and interventional studies should be performed under controlled conditions and assess varying types, intensities, and volumes of PA. Second, PA was assessed using the IPAQ-SF, a self-reported instrument reflecting activity over one week. Although validated, such measures are prone to recall and social desirability bias and may not capture habitual PA. Future studies should incorporate objective methods, such as accelerometers or wearable devices, over longer monitoring periods. Third, the analysis focused on PA without accounting for other behavioral and biological factors influencing IgG glycosylation, such as anthropometric measures, diet, sleep, and psychosocial factors.

Overall, the present findings do not support a robust association between self-reported PA and IgG N-glycosylation in young, apparently healthy adults. Rather than excluding a biological relationship, these results suggest that any such association may be modest, cumulative, and highly dependent on population characteristics and the method of physical activity assessment.
