# Supplementary material for: Physical activity and IgG N-glycosylation in medical students: a cross-sectional study
Source: Croat Med J. 2026 Jun;67(3):156–63. doi: 10.3325/cmj.2026.67.156 (PMC13247745; doi:10.3325/cmj.2026.67.156)
Supplement: Supplementary Figure 1 [file CroatMedJ_67_s013.pdf]

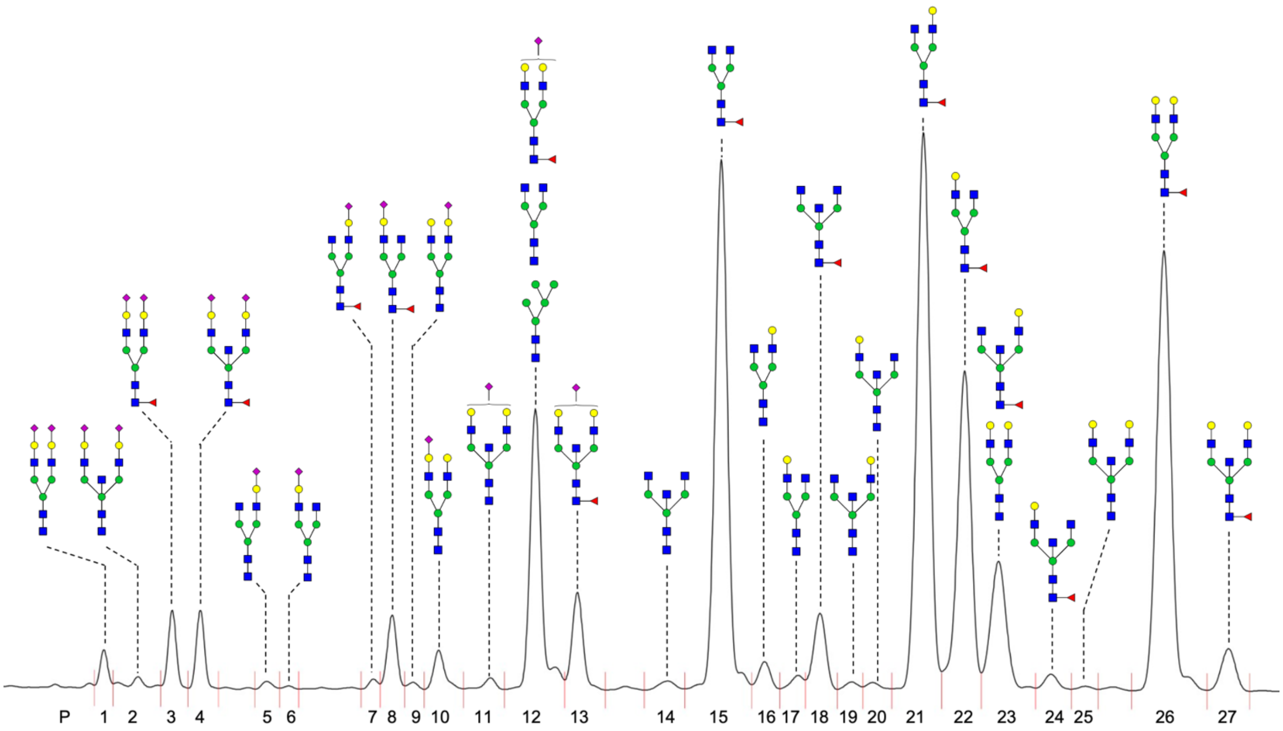

Supplemental Figure 1. Representative CGE-LIF electropherogram of IgG N-glycans showing 27 peaks corresponding to the IgG N-glycan structures defined in Supplemental Table 1.
