# Supplementary material for: Physical activity and IgG N-glycosylation in medical students: a cross-sectional study
Source: Croat Med J. 2026 Jun;67(3):156–63. doi: 10.3325/cmj.2026.67.156 (PMC13247745; doi:10.3325/cmj.2026.67.156)
Supplement: Supplementary Table 1 [file CroatMedJ_67_s014.pdf]

Supplemental Table 1. Structural annotation and nomenclature of IgG N-glycan peaks (P1–P27).

	Structure	
**P1**	Disialylated digalactosylated biantennary N-glycan (A2G2S2)	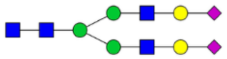
**P2**	Disialylated digalactosylated biantennary N-glycan with bisecting GlcNAc (A2BG2S2)	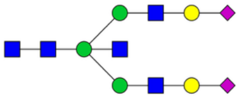
**P3**	Core-fucosylated disialylated digalactosylated biantennary N-glycan (FA2G2S2)	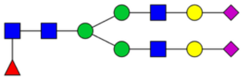
**P4**	Core-fucosylated disialylated digalactosylated biantennary N-glycan with bisecting GlcNAc (FA2BG2S2)	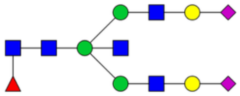
**P5**	Monosialylated monogalactosylated biantennary N-glycan (α1,6-arm) (A2[6]G1S1)	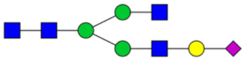
**P6**	Monosialylated monogalactosylated biantennary N-glycan (α1,3-arm) (A2[3]G1S1)	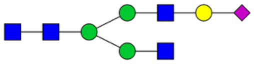
**P7**	Core-fucosylated monosialylated monogalactosylated biantennary N-glycan (α1,6-arm) (FA2[6]G1S1)	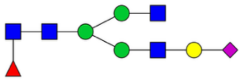
**P8**	Core-fucosylated monosialylated monogalactosylated biantennary N-glycan (α1,3-arm) (FA2[3]G1S1)	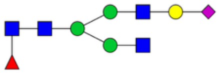
**P9**	Monosialylated digalactosylated biantennary N-glycan (α1,6-arm) (A2G2[6]S1)	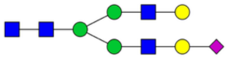
**P10**	Monosialylated digalactosylated biantennary N-glycan (α1,3-arm) (A2G2[3]S1)	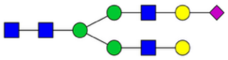
**P11**	Monosialylated digalactosylated biantennary N-glycan with bisecting GlcNAc (A2BG2S1)	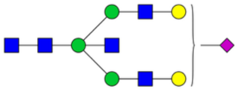
**P12**	Core-fucosylated monosialylated digalactosylated biantennary N-glycan (FA2G2S1)	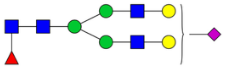
	High-mannose N-glycan (Man5) (M5)	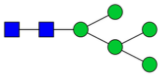

	Agalactosylated biantennary N-glycan (A2)	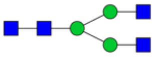
**P13**	Core-fucosylated monosialylated digalactosylated biantennary N-glycan with bisecting GlcNAc (FA2BG2S1)	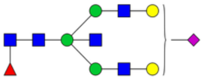
**P14**	Biantennary N-glycan with bisecting GlcNAc (A2B)	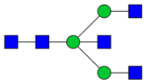
**P15**	Core-fucosylated biantennary N-glycan (FA2)	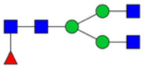
**P16**	Monogalactosylated biantennary N-glycan (α1,6-arm) (A2[6]G1)	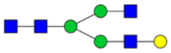
**P17**	Monogalactosylated biantennary N-glycan (α1,3-arm) (A2[3]G1)	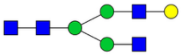
**P18**	Core-fucosylated biantennary N-glycan with bisecting GlcNAc (FA2B)	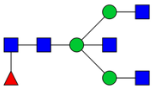
**P19**	Monogalactosylated biantennary N-glycan with bisecting GlcNAc (α1,6-arm) (A2B[6]G1)	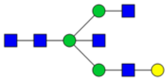
**P20**	Monogalactosylated biantennary N-glycan with bisecting GlcNAc (α1,3-arm) (A2B[3]G1)	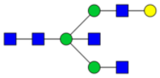
**P21**	Core-fucosylated monogalactosylated biantennary N-glycan (α1,6-arm) (FA2[6]G1)	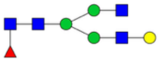
**P22**	Core-fucosylated monogalactosylated biantennary N-glycan (α1,3-arm) (FA2[3]G1)	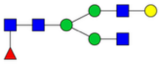
**P23**	Core-fucosylated monogalactosylated biantennary N-glycan (α1,6-arm) (FA2[6]G1)	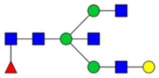
	Digalactosylated biantennary N-glycan (A2G2)	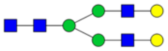
**P24**	Core-fucosylated monogalactosylated biantennary N-glycan (α1,3-arm) (FA2B[3]G1)	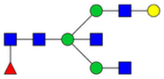
**P25**	Digalactosylated biantennary N-glycan with bisecting GlcNAc (A2BG2)	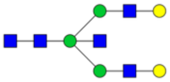
**P26**	Core-fucosylated digalactosylated N-glycan (FA2G2)	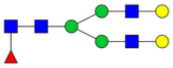

**P27**	Core-fucosylated digalactosylated biantennary N-glycan with bisecting GlcNAc (FA2BG2)	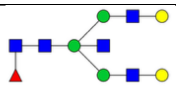

Note: Structure abbreviations: all N-glycans have two core GlcNAcs; F at the start of the abbreviation indicates a core-fucose α1,6-linked to the inner GlcNAc; Mx, number (x) of mannose on core GlcNAcs; Ax, number of antenna (GlcNAc) on trimannosyl core; A2, biantennary with both GlcNAcs as β1,2-linked; B, bisecting GlcNAc linked β1,4 to β1,3 mannose; Gx, number (x) of β1,4-linked galactose on antenna; Sx, number (x) of α2,6- linked sialic acids linked to galactose
