# Supplementary material for: Physical activity and IgG N-glycosylation in medical students: a cross-sectional study
Source: Croat Med J. 2026 Jun;67(3):156–63. doi: 10.3325/cmj.2026.67.156 (PMC13247745; doi:10.3325/cmj.2026.67.156)
Supplement: Supplementary Table 2 [file CroatMedJ_67_s015.pdf]

Supplemental Table 2. Raw data containing the numerical values of all quantified IgG N-glycan peaks (P1–P27) for all study participants.

P1	P2	P3	P4	P5	P6	P7	P8	P9	P10	P11	P12	P13	P14	P15	P16	P17	P18	P19	P20	P21	P22	P23	P24	P25	P26	P27	G0	G1	G2	S	S1	S2	B	CF
0,484173	0,313537	2,420993	2,239967	0,23511	0,046221	0,273527	2,289632	0,260663	0,759348	0,339837	15,21107	2,394851	0,277449	8,572341	0,646249	0,319162	1,387201	0,248395	0,200832	16,68871	9,753858	5,671956	0,496057	0,180479	25,95268	1,951963	10,2764254	34,1562928	28,1933077	27,3739741	21,8942767	5,47969744	15,763011	95,6719374
0,332852	0,180234	1,797364	1,66901	0,173273	0,025225	0,248297	1,954793	0,231444	0,49778	0,299224	11,91176	2,353726	0,290871	11,60996	0,382996	0,230464	2,05247	0,213722	0,249941	18,39285	11,12128	5,488828	0,592634	0,155219	25,20443	1,884262	14,0170937	36,8403744	27,3684586	21,7740732	17,77642	3,99765323	15,5006813	96,721835
0,290709	0,156058	1,327188	1,026848	0,235526	0,021697	0,21336	1,336431	0,200365	0,3596	0,152575	9,63012	1,535547	0,322392	16,38071	0,2906	0,256072	4,996159	0,290221	0,344815	19,01492	9,233799	6,302304	0,632443	0,106366	23,21724	1,670551	21,7985263	36,5315353	25,108496	16,5614424	13,7478262	2,81361612	17,6165016	96,959157
0,338153	0,215258	1,834074	1,507669	0,163149	0,052984	0,316248	2,60475	0,282735	0,565206	0,302672	12,31229	1,941121	0,341162	13,87392	0,592804	0,329153	2,094949	0,249391	0,388416	20,34438	11,17009	5,053383	0,599285	0,130083	20,83523	1,406885	16,3352805	38,7868518	22,4068258	22,4710419	18,5698584	3,90118347	14,2523006	96,0427173
0,434656	0,286757	2,198093	2,118851	0,133269	0,074096	0,267289	2,683329	0,393426	0,705423	0,2827	10,06935	3,098036	0,283193	18,57657	0,648939	0,376004	2,486549	0,283464	0,249264	17,79727	12,37772	4,918825	0,650674	0,165181	16,75441	1,664593	21,351024	37,3104005	18,5882861	22,7502895	17,710821	5,03946843	16,4917266	95,6826759
0,36798	0,21455	1,886295	1,969318	0,159644	0,016614	0,317523	2,21598	0,371785	0,498265	0,288621	13,34635	2,38608	0,24913	13,65379	0,228422	0,120707	2,527193	0,221983	0,233765	18,77869	9,577168	6,08195	0,628486	0,140684	21,30256	2,024276	16,4617511	35,9402474	23,5127098	24,0852917	19,6386032	4,44668851	16,9987057	96,8818588
0,317839	0,168889	1,506308	1,388597	0,159748	0,030988	0,23918	2,377068	0,243671	0,425927	0,253648	11,65069	2,053604	0,281121	12,61973	0,319591	0,265841	2,1865	0,200935	0,289236	18,02795	12,97323	5,188523	0,640564	0,141623	23,84486	1,801728	15,1483131	38,0590228	25,8924027	20,9002615	17,504966	3,39529545	14,6539353	96,8884223
0,390859	0,209398	3,374957	1,700007	0,182762	0,037841	0,248374	2,312572	0,238341	0,500417	0,270313	11,97251	2,365064	0,275434	16,02689	0,267778	0,244056	2,023076	0,178654	0,23831	18,72474	12,30197	3,885566	0,475255	0,127011	20,23244	1,320365	18,3025304	36,2710061	21,6527554	23,7737081	18,1055699	5,66813821	13,0521421	96,842771
0,47885	0,292073	3,168413	2,090796	0,169234	0,084167	0,336698	2,415511	0,287151	0,844761	0,410115	14,68678	2,45382	0,290216	11,88622	0,853152	0,424412	2,00831	0,285998	0,232098	17,69353	9,716171	5,068685	0,517157	0,185074	21,44872	1,590362	14,1963153	34,8195951	23,243101	27,7409886	21,7059359	6,03505267	15,4372896	95,1587503
0,349286	0,19501	1,897592	1,597496	0,159147	0,041441	0,276911	2,172445	0,284319	0,662284	0,284279	11,14619	2,290231	0,364383	17,52126	0,603443	0,333298	2,917999	0,25751	0,354436	18,88278	11,00168	5,107249	0,683687	0,143844	18,81518	1,496324	20,8370382	37,2838558	20,4881894	21,3909165	17,345048	4,04586857	15,7176426	95,9608446
0,363234	0,169313	2,39758	1,612141	0,158858	0,02976	0,256544	1,793232	0,262856	0,501356	0,297829	9,970024	2,374479	0,276662	17,35537	0,418954	0,258432	1,923809	0,206059	0,275861	21,13078	11,50834	4,588894	0,542627	0,149656	19,5722	1,544727	19,5676648	38,9534845	21,2794406	20,1994101	15,654396	4,54501404	13,9704969	96,6291332
0,351253	0,230484	2,149979	1,816168	0,152939	0,045578	0,257014	2,310852	0,268218	0,593486	0,276436	8,440197	2,390249	0,411889	24,07223	0,518069	0,293142	3,482166	0,284623	0,343464	19,8695	11,80563	4,170908	0,567707	0,131924	13,85962	1,119789	27,9067029	37,7723921	15,0791353	19,2417697	14,7035749	4,53819471	15,1933675	96,1068069
0,336102	0,150167	1,627795	1,383072	0,133081	0,031714	0,243866	1,777219	0,223106	0,479653	0,260616	9,304602	2,189327	0,311137	18,09609	0,403909	0,252801	3,01653	0,239695	0,249093	20,13968	11,71594	5,012132	0,642946	0,144274	19,91334	1,521606	21,4667972	38,7338621	21,6225728	18,176768	14,6726059	3,50416209	15,150974	96,7781923
0,591905	0,182089	1,939053	1,51529	0,15507	0,041159	0,246045	1,942274	0,264178	0,532448	0,264817	12,11159	2,151294	0,246531	17,6501	0,41776	0,248326	3,011204	0,276167	0,396255	16,45939	10,12708	5,093705	0,549463	0,341938	21,28556	1,659656	20,9706772	33,6690333	23,3571482	22,0031413	17,7620958	4,24104554	15,7355603	96,0294588
0,322343	0,192252	2,523384	2,048516	0,145687	0,027812	0,249285	1,837313	0,272806	0,531447	0,30554	10,90327	2,956879	0,302752	14,56774	0,359956	0,207472	2,088408	0,199214	0,310855	20,60179	10,6793	4,811121	0,56369	0,141428	20,94815	1,754247	16,9839281	37,7890764	22,8775326	22,3494629	17,2554612	5,09400164	15,6980309	96,6755377
0,360265	0,233296	2,613449	2,307501	0,192024	0,027917	0,281541	2,1848	0,286869	0,567009	0,331038	15,57099	2,752546	0,249751	7,800981	0,293942	0,200632	1,972384	0,209063	0,175289	15,7686	9,295628	5,679634	0,551161	0,159938	27,18048	2,32226	10,0665034	32,3132239	29,7910797	27,829193	22,2908105	5,5383825	17,0172074	96,6987368
0,358763	0,21662	2,686981	1,93853	0,153243	0,015954	0,295625	2,493687	0,268846	0,48277	0,302686	11,94782	2,429507	0,261022	14,3778	0,273477	0,175185	1,752772	0,173143	0,284533	20,43899	11,33254	4,376061	0,53383	0,126782	20,90175	1,443522	16,3846383	37,5718172	22,4625199	23,5810246	18,3823361	5,1986885	13,8331395	96,9082877
0,478707	0,259759	2,545172	2,091548	0,173935	0,027502	0,259336	2,435204	0,281631	0,563111	0,259486	11,03473	2,562501	0,332814	15,54021	0,351957	0,222908	2,759642	0,216837	0,247703	18,70874	11,52772	4,906414	0,691716	0,140426	19,75737	1,591961	18,6384345	36,8854156	21,4964161	22,9797338	17,6028836	5,3768502	16,0657817	96,4421229
0,390841	0,237731	2,432163	2,735772	0,154997	0,020526	0,223329	2,201347	0,343791	0,679054	0,368289	8,803499	4,121237	0,420464	20,01627	0,265404	0,185959	2,579509	0,250002	0,336318	18,78903	11,96968	4,575619	0,683834	0,169846	15,16007	1,868001	23,0202506	37,0623024	17,2009135	22,7165335	16,9190168	5,79751677	18,3498183	96,1761113
0,441432	0,197863	2,780321	2,007951	0,153646	0,039956	0,27175	2,495525	0,285728	0,65134	0,274296	14,1202	2,374933	0,348738	13,86202	0,40504	0,270366	1,870044	0,208373	0,358203	17,43833	10,92333	4,645201	0,539881	0,147027	21,18563	1,618745	16,0943377	34,8180192	22,9707271	26,116916	20,6847785	5,43213748	14,6035395	96,2148084
0,314783	0,197436	2,05952	2,500145	0,183984	0,026745	0,195779	1,812611	0,284041	0,583793	0,353912	8,518282	4,021786	0,390943	16,31882	0,256476	0,200242	2,361006	0,247293	0,285499	20,12556	12,53792	5,008981	0,793399	0,19634	17,80098	2,163537	19,1205161	39,5582967	20,2134482	21,1077391	16,0226244	5,08511461	18,5685909	96,4693269
0,458969	0,228323	3,036701	1,736377	0,146945	0,03013	0,292635	2,217286	0,2323	0,571938	0,288368	12,27051	2,011513	0,273164	15,51004	0,367304	0,228768	1,662882	0,19003	0,283818	21,08316	10,7266	3,918657	0,468377	0,132959	20,56054	1,202388	17,4233167	37,2180738	21,8673147	23,4912948	18,0380512	5,45324355	12,3806767	96,5714634
0,452889	0,190762	1,949088	1,445479	0,165117	0,053391	0,252726	1,655344	0,213172	0,830906	0,283656	10,99035	2,244151	0,231588	13,32826	0,74116	0,382808	1,769462	0,242887	0,24426	20,91911	10,78203	5,331712	0,484063	0,173294	22,77596	1,503071	15,3852068	39,2706969	24,5414843	20,802612	16,7496696	4,05294234	14,1959598	95,7787746
0,502449	0,283473	2,906504	2,313766	0,19241	0,024027	0,310888	2,396543	0,262058	0,592897	0,327514	14,33593	2,406105	0,293307	12,02796	0,311165	0,155425	1,92744	0,182591	0,271495	18,5592	9,727819	4,589971	0,509545	0,126926	22,81938	1,620878	14,2518877	34,3148711	24,5726756	26,8605656	20,8530318	6,00753378	14,8563286	96,4734764
0,405243	0,264347	2,115489	1,83561	0,178651	0,038286	0,256773	1,77126	0,255128	0,603115	0,397247	12,81724	2,494961	0,329434	9,932397	0,647611	0,320877	2,025793	0,268444	0,328682	17,54736	9,45881	6,191131	0,537447	0,183376	25,9708	2,229111	12,3612193	35,5117904	28,5532882	23,5737022	18,9253377	4,6483645	17,1879149	95,7542808
0,435594	0,183277	2,188675	1,661489	0,15541	0,054723	0,250203	1,822247	0,262086	0,620021	0,25178	11,88435	2,383153	0,269847	14,07392	0,623165	0,336193	2,089408	0,213276	0,215907	18,39716	10,15311	5,251995	0,56094	0,124547	23,49752	1,73124	16,4840711	35,8624816	25,4318308	22,2216165	17,7387412	4,48287529	14,9831194	96,2425739
0,467693	0,218121	3,149274	1,949519	0,153109	0,043843	0,262165	2,063268	0,267857	0,707542	0,278236	13,80043	2,469784	0,275729	12,82669	0,574699	0,280265	1,6188	0,191373	0,261435	19,40641	9,714936	4,557151	0,523539	0,137045	22,2243	1,523214	14,7291089	35,5288446	23,8973609	25,8446855	20,0569777	5,78770782	14,0114535	96,1409861
0,292387	0,174358	1,888731	1,32223	0,144301	0,02607	0,243962	1,976823	0,218951	0,462435	0,206825	11,61323	1,9052	0,285616	13,34694	0,461408	0,244096	1,848982	0,160965	0,292865	19,73175	11,28492	4,35128	0,499865	0,105208	25,18699	1,426663	15,5276463	37,1374262	26,7984405	20,5364869	16,8478279	3,68865905	12,6175226	96,915355
0,368881	0,242462	2,298881	2,299312	0,192013	0,052449	0,292146	2,835891	0,325513	0,584194	0,302641	12,36099	2,630846	0,339825	13,85833	0,443166	0,272383	1,916208	0,211862	0,310763	19,84139	11,55616	4,897433	0,573916	0,133686	19,36825	1,509471	16,1112888	38,099809	21,0074064	24,7814958	19,5729544	5,20854139	15,3654955	96,2208836
0,360552	0,184227	2,004425	1,520543	0,122619	0,03686	0,225623	2,324072	0,240425	0,562393	0,27788	11,15581	2,448875	0,27083	13,68206	0,445505	0,28101	2,24811	0,214709	0,306671	18,32271	13,28355	4,522196	0,652921	0,140239	22,26244	1,614177	16,2478902	38,1393302	24,086359	21,5264207	17,4448954	4,08152528	14,4430558	96,5461129
0,336761	0,185695	2,534031	1,944662	0,163417	0,018737	0,321901	2,365612	0,277139	0,430297	0,252648	11,91859	2,384663	0,238217	14,70316	0,23033	0,150099	1,968565	0,174921	0,20246	19,89681	11,33789	4,35906	0,533407	0,11117	21,5002	1,495559	16,9038567	36,8716999	23,098613	23,1258305	18,1264815	4,99934899	13,8460453	97,2291059
0,352886	0,276334	2,577486	2,704662	0,311018	0,022051	0,310696	1,792251	0,338184	0,454529	0,404484	16,65806	3,032583	0,29006	7,744824	0,183088	0,151184	2,036167	0,161735	0,212181	16,36388	7,758115	5,832941	0,533451	0,178709	26,54187	2,408617	10,1082456	31,3117861	29,2367698	29,3431985	23,4099988	5,93319967	18,1386678	96,6512331
0,358338	0,204798	1,87441	1,645031	0,176556	0,044613	0,223716	1,775064	0,257151	0,551671	0,316754	11,90397	2,569563	0,306107	10,72272	0,595056	0,313843	1,785587	0,258956	0,232741	18,27483	10,67404	6,014496	0,576557	0,186656	25,45514	2,130489	12,8880208	37,1527192	27,9318167	22,0274433	17,9214148	4,10602845	16,3209527	96,1749119
0,347578	0,240188	1,647393	1,848405	0,165529	0,022543	0,28231	1,981052	0,246004	0,416038	0,259169	9,934974	2,656742	0,236846	14,43517	0,248654	0,164377	2,304707	0,196904	0,193188	20,90475	12,03689	5,109349	0,617498	0,145624	21,36804	1,787708	17,0111496	39,5516489	23,3486245	20,088577	15,9967333	4,09184372	15,6279541	97,1115128
0,394525	0,234334	2,120008	1,672185	0,116342	0,021194	0,239084	2,11787	0,260275	0,49819	0,217321	9,640458	2,389345	0,267509	17,49748	0,269568	0,211289	2,424739	0,224067	0,252667	20,52708	12,04417	4,511729	0,555002	0,143221	19,51125	1,580827	20,2015008	38,6180747	21,2476795	19,932745	15,5091152	4,42362981	14,4813839	96,8876837
0,337772	0,203292	1,932722	1,499876	0,193153	0,037374	0,273241	2,293512	0,290208	0,474156	0,426805	15,95501	1,948508	0,240771	6,472499	0,388868	0,219321	1,50269	0,189445	0,209833	15,09912	9,592035	6,060463	0,66502	0,183911	29,92236	2,654321	8,27668816	32,6637685	33,0027364	26,056807	22,0537742	4,00303277	15,9016072	96,5799985
0,395566	0,296751	2,654693	1,838757	0,215047	0,069473	0,281601	2,129016	0,255098	0,555762	0,300468	15,78952	1,985702	0,308915	7,322306	0,400292	0,305366	1,300411	0,225506	0,261371	15,86785	10,21105	4,776621	0,501454	0,15504	29,31549	1,833211	8,97179484	32,6958799	31,4445019	26,8878234	21,6787378	5,20908562	13,8461909	96,2385071
0,426426	0,182285	2,566699	1,617713	0,186401	0,024113	0,265736	2,09608	0,205871	0,595401	0,277584	11,54608	2,409916	0,302791	12,77534	0,370428	0,247423	1,12141	0,161308	0,280333	20,17244	12,83906	3,723449	0,455322	0,140761	23,4153	1,412698	14,2253778	38,3193639	25,0141909	22,4410674	17,6392231	4,80184429	12,1075593	96,5926877
0,433429	0,222639	1,877459	1,75783	0,160512	0,027116	0,273645	2,605617	0,288557	0,480854	0,281202	10,51436	2,147841	0,319186	16,21783	0,296429	0,217408	2,831882	0,246182	0,242395	18,77315	12,48653	5,522613	0,646394	0,14906	19,20719	1,668572	19,3890833	38,471155	21,0467361	21,0930257	16,7971941	4,29583163	16,05251	96,631523
0,415444	0,216125	2,432282	1,399202	0,154479	0,051161	0,258967	2,487488	0,258047	0,690814	0,291381	13,96702	1,745497	0,298001	13,38796	0,655661	0,332903	1,849385	0,218865	0,331268	16,74184	10,98001	4,509325	0,492384	0,135262	24,06535	1,39641	15,5723226	34,3438078	25,6579545	24,425915	19,9522394	4,47367561	12,9137694	95,9409504
0,456807	0,254288	2,070446	2,048173	0,198706	0,033697	0,2701	2,223542	0,282063	0,653202	0,331288	13,58664	2,569394	0,305349	11,54526	0,401983	0,220571	2,044214	0,230142	0,24684	17,7906	10,48155	5,449011	0,575322	0,174118	23,35403	1,882599	13,9394402	35,5096707	25,4923382	25,0585509	20,2133298	4,84522107	16,1624668	96,1987788
0,433227	0,239774	2,710192	2,328214	0,162743	0,033448	0,274962	3,409851	0,356833	0,667291	0,288429	12,28219	2,759843	0,311298	16,03001	0,410074	0,196349	1,946235	0,211982	0,245946	17,35247	12,76676	4,492063	0,631701	0,124975	17,97032	1,502505	18,2620313	36,2566996	19,5704675	25,9108015	20,2073613	5,70344021	15,061925	96,3227676
0,533619	0,267496	2,564649	1,806536	0,165254	0,036813	0,277388	1,812877	0,25771	0,666585	0,335325	13,67765	2,255912	0,303525	11,15813	0,475703	0,255594	1,938441	0,219597	0,280685	18,6238	9,285234	4,957005	0,529117	0,155416	25,17806	1,669485	13,4420875	34,7352452	27,0875787	24,7350885	19,5465794	5,1885091	14,764664	96,0342873
0,411022	0,182411	2,561909	1,849195	0,158035	0,017725	0,281439	3,279068	0,277718	0,444659	0,273051	13,26343	2,145277	0,203224	14,5204	0,203409	0,145327	2,102977	0,168384	0,199751	18,25004	12,74406	4,025554	0,557019	0,117497	20,39542	1,372123	16,8013781	36,2391377	21,8522359	25,1072483	20,1102133	4,99703499	13,1766796	97,2019873
0,578903	0,323469	2,147705	1,719657	0,158018	0,106797	0,292276	1,962536	0,211404	0,897506	0,360046	11,90012	2,200953	0,293253	13,25666	1,385015	0,686887	2,113213	0,292998	0,19899	19,6449	10,32956	5,158731	0,495084	0,178784	21,50629	1,358332	15,7011087	38,2847871	23,099281	22,9148232	18,1335228	4,78130048	14,7291407	94,3141755
0,370064	0,205162	1,636112	1,69901	0,140205	0,046506	0,259929	1,83011	0,248276	0,518424	0,238831	10,2146	2,008339	0,338207	13,44616	0,660796	0,341709	2,549508	0,262622	0,314654	21,55756	10,54513	6,403821	0,591857	0,134651	21,50806	1,633115	16,3824645	40,7991509	23,3450612	19,4733235	15,5513442	3,9219793	16,4285014	96,1685308
0,355234	0,194001	1,98463	1,62874	0,218657	0,033264	0,290959	1,691295	0,223685	0,463635	0,298639	10,88727	2,195568	0,263773	14,6458	0,344311	0,305873	2,062931	0,169552	0,221262	20,96189	10,18288	4,590511	0,448786	0,120287	23,47239	1,536309	17,0078526	37,3026061	25,1813317	20,5082095	16,3369336	4,17127591	13,7589575	96,7811354
0,485338	0,212349	2,350436	1,413484	0,163082	0,023299	0,283811	2,634312	0,236085	0,540855	0,280415	13,21971	1,735611	0,270814	13,84369	0,264217	0,181077	1,501506	0,158683	0,273906	18,90271	12,45204	3,729113	0,490262	0,11182	22,93059	1,258177	15,6242346	36,4711939	24,3133764	23,5911951	19,1272389	4,46395616	11,4421592	96,7963761
0,432468	0,269092	2,578643	1,777872	0,177996	0,039701	0,357996	2,568972	0,323734	0,616182	0,298895	12,05385	2,084326	0,329392	15,80337	0,509232	0,283238	1,949227	0,208367	0,326034	21,59139	10,43115	4,54092	0,465021	0,146918	18,73833	1,225346	18,0589344	38,3064499	20,0849487	23,5496669	18,4980418	5,05162512	13,6040407	96,043803
0,289564	0,149871	3,278123	2,005822	0,146586	0,007943	0,244251	2,178053	0,298034	0,486381	0,246279	11,13958	3,087828	0,379345	17,07309	0,063066	0,105432	2,12224	0,157855	0,360506	20,07658	11,70915	4,158405	0,611109	0,107213	18,00759	1,616022	19,5539656	37,2026998	19,7099484	23,5333862	17,8160622	5,71732406	14,9866206	97,2048847
0,581149	0,330701	2,290164	1,798865	0,203834	0,095989	0,280157	2,447262	0,226908	0,94822	0,492306	13,2568	2,280511	0,36503	11,55872	1,070029	0,604526	1,755894	0,272139	0,310286	16,56644	11,50466	5,428811	0,572444	0,200602	22,60515	1,561521	13,7333279	36,4718962	24,4628949	25,3318809	20,3113777	5,02050325	15,4294193	94,2759051
0,295971	0,164806	1,806762	1,756581	0,131953	0,020906	0,215355	1,881145	0,218143	0,385387	0,212257	8,460104	2,370571	0,346949	17,31236	0,369092	0,24156	2,719807	0,227357	0,385452	20,93715	12,85236	4,815636	0,546038	0,118155	19,62018	1,449456	20,4073804	40,430644	21,2171765	17,9447991	13,915097	4,02970208	15,134028	96,8776853
0,52027	0,332507	3,245671	2,006491	0,189673	0,04545	0,264637	1,990972	0,238693	0,699571	0,379545	13,54846	2,666773	0,3405	11,80651	0,61844	0,352553	1,495763	0,222741	0,265234	18,02864	10,63919	4,81973	0,467649	0,161405	22,69901	1,778743	13,6667121	35,4763259	24,6823935	26,1745684	20,0589164	6,11565207	14,9632917	95,6257558
0,444303	0,236404	2,384621	2,004171	0,156504	0,018935	0,211442	2,290213	0,245299	0,545028	0,335726	11,89004	3,008214	0,316513	13,21864	0,17433	0,198215	2,259756	0,188538	0,268959	15,70792	12,58522	4,844688	0,746432	0,181095	23,02147	2,142888	15,8542762	34,8447764	25,4407103	23,8602372	18,771685	5,08855217	16,5955237	96,6777114
0,392653	0,216329	1,728298	1,17448	0,129138	0,035491	0,231354	1,961572	0,225181	0,558312	0,171054	11,43579	1,625674	0,240656	15,44732	0,374792	0,226077	1,870385	0,179435	0,279381	18,35368	11,80745	4,66592	0,528268	0,156523	24,16555	1,498212	17,6149124	36,5322766	25,9034419	19,9493691	16,4262994	3,5230697	12,6469167	96,8047202
0,345142	0,170123	2,23212	1,7582	0,158621	0,018901	0,26901	2,481494	0,298262	0,521293	0,260374	11,2939	2,40545	0,313941	17,35406	0,272284	0,17967	1,845794	0,173514	0,296679	19,53404	12,21309	4,006044	0,570792	0,11303	19,60478	1,335427	19,508715	37,2364208	21,047757	22,2071072	17,7026945	4,50441274	13,2459209	96,8789795
0,505573	0,280104	2,885038	2,540003	0,215516	0,024766	0,284308	2,093305	0,305389	0,627982	0,320803	15,87395	2,868767	0,260532	8,85845	0,309361	0,179315	2,138504	0,204592	0,225808	15,58501	8,835692	5,617329	0,537735	0,141167	25,72817	2,23377	11,2935184	31,595653	28,1930615	28,9177671	22,6871691	6,23059801	17,4247112	96,3875646
0,446924	0,232762	1,466823	1,733788	0,162633	0,032573	0,28075	2,29733	0,267677	0,544642	0,307345	9,226049	2,30142	0,299537	18,12487	0,474587	0,265333	3,414521	0,302872	0,230663	19,71809	11,97818	5,643508	0,659638	0,145596	17,81496	1,517479	21,8628626	39,3158949	19,4993784	19,321864	15,4373164	3,88454766	16,8075237	96,2827871
0,364434	0,199611	1,747429	1,492097	0,126558	0,054348	0,269411	2,166906	0,258907	0,565233	0,267813	10,83378	2,046281	0,306838	16,0101	0,665979	0,375088	2,422926	0,249541	0,31372	20,76089	11,19659	4,842496	0,538799	0,129336	20,25701	1,394343	18,7668039	38,9990763	21,8119957	20,4221241	16,6130856	3,80903854	14,2242172	96,117019
0,445619	0,267526	2,086936	2,102666	0,177808	0,056451	0,282188	2,388734	0,29587	0,705474	0,318612	12,84167	2,574821	0,308882	11,46758	0,738847	0,400522	2,069045	0,300899	0,234193	17,68947	10,86791	5,808154	0,568335	0,181185	22,54506	1,93975	13,8921517	36,7316789	24,7490996	24,6270698	19,7078045	4,91926522	16,7302459	95,5531812
0,446472	0,203534	1,723856	1,493729	0,13946	0,038129	0,237158	1,931851	0,191991	0,532198	0,270279	9,625559	2,088809	0,338186	16,98066	0,658695	0,362038	2,269464	0,23381	0,333595	21,28339	12,53616	4,560866	0,568955	0,142599	19,37901	1,30149	19,6134252	40,5894924	20,849795	18,9472874	15,0747386	3,87254873	13,823018	96,1040256
0,423832	0,223921	2,248258	1,72578	0,155411	0,044304	0,303899	1,872654	0,233137	0,675488	0,241907	14,23701	1,898573	0,199829	11,15161	0,738874	0,298157	1,671327	0,223345	0,180451	19,21422	8,793312	5,28879	0,431534	0,133567	25,53627	1,592886	13,0569254	35,2609488	27,334246	24,3478799	19,7139653	4,63391451	13,8481449	96,2178822
0,450343	0,209847	1,840139	1,477721	0,139193	0,042057	0,25662	2,00815	0,277519	0,545648	0,271874	9,763843	2,054129	0,410401	18,54908	0,449718	0,284438	3,39161	0,264348	0,411025	19,84471	10,77742	5,278621	0,638347	0,130589	18,63099	1,446935	22,3857183	38,0074226	20,2398199	19,3670392	15,3828264	3,98421278	16,0102118	96,1069787
0,303333	0,16576	1,93282	1,612654	0,157602	0,026263	0,310875	2,622607	0,315463	0,473085	0,242961	10,71683	1,945277	0,369552	19,69302	0,233961	0,183479	2,443087	0,197532	0,314621	19,60932	11,52634	4,507713	0,570427	0,10417	18,12919	1,359616	22,4904677	37,1183092	19,5797509	20,8114722	16,7996168	4,01185539	13,8240287	96,914304
0,394124	0,232624	1,518206	1,869931	0,166906	0,059336	0,292583	1,667468	0,264995	0,723088	0,271763	12,38424	2,332914	0,338394	9,77232	1,105188	0,45731	1,77061	0,340522	0,295473	20,62732	9,421439	7,127459	0,555398	0,170798	23,34042	1,973814	11,9440736	40,1409963	25,619624	22,2953061	18,259217	4,0360891	17,3709614	95,1540189
0,386713	0,239035	2,356279	1,856439	0,153526	0,032135	0,243481	2,224994	0,32078	0,56167	0,296212	12,78752	2,402567	0,31367	13,35578	0,323637	0,206895	2,689884	0,22777	0,239736	16,84518	11,13957	5,136261	0,628634	0,148039	22,83284	1,80324	16,3999252	34,8338982	24,8456197	23,9205569	19,070086	4,85047095	16,0211406	96,5416216
0,381153	0,185551	2,022402	1,612168	0,132617	0,035473	0,250242	2,339404	0,222003	0,490833	0,250618	10,42478	2,088608	0,278714	16,05976	0,483412	0,260206	1,806473	0,185887	0,264594	21,03352	12,75743	4,188806	0,497715	0,11951	20,30581	1,301137	18,1487917	39,679974	21,7310556	20,4401787	16,2380151	4,20216364	12,78249	96,7087316
0,319483	0,201202	1,900641	1,653878	0,24494	0,0484	0,413828	2,60412	0,251104	0,519998	0,603653	12,3097	2,210719	0,294492	14,05972	0,444718	0,352571	1,821063	0,218753	0,22779	17,2361	12,40338	4,723178	0,526483	0,237747	22,3291	1,592233	16,2159829	36,2238973	24,2198712	23,3402487	19,254789	4,0854597	14,3472045	96,0251724
0,488085	0,279244	2,956365	2,227545	0,163318	0,064978	0,247664	1,671692	0,302751	0,872609	0,328602	10,74995	2,972926	0,367111	17,83892	0,769323	0,410598	2,441808	0,312052	0,335392	19,95591	9,372027	5,11472	0,526323	0,171634	17,5243	1,502941	20,6542893	36,8078362	19,2048676	23,3330069	17,3799098	5,95309712	16,5854747	95,1327837
0,413844	0,27959	3,043147	2,196029	0,204499	0,028661	0,288484	2,631607	0,280764	0,508647	0,234178	14,97153	2,724222	0,260295	9,28301	0,290393	0,194707	1,624617	0,152998	0,2047	15,68787	11,52688	4,583302	0,541063	0,142784	25,59509	1,911703	11,1897847	33,2468769	27,7037057	27,8596328	21,9154096	5,94422323	14,8845614	96,7976814
0,659446	0,324801	2,016525	1,933459	0,146989	0,083544	0,307917	2,494161	0,367298	0,825465	0,197771	9,723036	2,392271	0,287434	21,59957	0,538905	0,316579	3,267784	0,292294	0,240217	18,59442	10,53312	5,019736	0,648479	0,134793	15,65396	1,474139	25,136163	36,1569514	17,2501045	21,4567812	16,5262046	4,93057664	16,2011712	95,587736
0,43878	0,1657	2,298112	1,485095	0,203145	0,020632	0,282464	2,459061	0,225226	0,562839	0,343756	15,16702	1,834177	0,207999	10,42199	0,284492	0,178201	1,523495	0,142754	0,213472	16,89427	11,28724	4,052521	0,44813	0,129398	26,90692	1,542079	12,1877373	33,5954943	28,6589363	25,5578321	21,1577794	4,40005261	12,1226455	96,8748255
0,447224	0,223612	1,849189	1,454417	0,162064	0,04942	0,210862	2,236631	0,199007	0,601695	0,26337	10,41132	1,956122	0,259522	14,46811	0,565489	0,36329	2,581862	0,237382	0,266072	18,824	12,91708	5,197545	0,590576	0,145885	21,77534	1,47509	17,3559772	39,0660618	23,459142	20,118819	16,1337039	3,98511507	14,6907974	96,2058059
0,346633	0,154426	1,806007	1,531825	0,155298	0,040321	0,283549	2,347972	0,248701	0,490382	0,279243	9,799667	1,826377	0,296884	16,56122	0,534024	0,34733	2,248805	0,251964	0,250331	20,62878	12,42823	5,055829	0,556336	0,140279	19,89042	1,401303	19,1256225	40,0920567	21,4530007	19,3293201	15,4866676	3,84265242	14,0073106	96,4607189
0,374893	0,219723	2,870377	2,143343	0,138372	0,015821	0,286263	1,889865	0,284815	0,56565	0,312697	9,499796	3,07321	0,363455	18,74248	0,156762	0,126894	1,858417	0,186844	0,30606	23,4714	10,87778	3,916242	0,516181	0,138611	16,46886	1,395484	20,9224445	39,4790924	17,9669641	21,6314991	16,0343733	5,59712584	14,4014215	96,8157812
0,427135	0,226372	2,140164	2,141811	0,172375	0,020911	0,248187	2,398537	0,300968	0,551341	0,301441	13,37048	2,895077	0,319283	11,24471	0,254015	0,173479	1,804339	0,183132	0,226474	15,96377	11,18708	5,065055	0,673346	0,150436	24,8912	2,251778	13,4243269	33,8676163	27,4077341	25,3003227	20,3441684	4,95615433	16,3065591	96,6787846
0,528967	0,227164	1,464169	1,820636	0,128523	0,036803	0,21319	2,149786	0,257999	0,626774	0,308711	9,292813	3,17545	0,329599	14,73494	0,282301	0,258958	2,126505	0,198668	0,317336	19,35848	13,34493	4,908589	0,742793	0,171733	20,60161	1,977574	17,2626839	39,5762914	22,8457306	20,3152942	16,2575184	4,05777576	16,3727052	96,3111565
0,487739	0,208339	2,090164	1,473758	0,13603	0,04021	0,217536	2,535295	0,222593	0,560434	0,263761	8,61544	2,117846	0,382637	20,90964	0,53553	0,324455	2,167709	0,234636	0,298949	19,85969	14,62369	4,009076	0,589323	0,142953	15,94433	1,108037	23,4365999	40,4349934	17,1781744	18,9502322	14,6944804	4,25575178	12,9840649	96,1655633
0,365971	0,377209	2,933235	1,832128	0,178754	0,032181	0,271468	2,545111	0,253152	0,447021	0,215994	14,76943	2,144644	0,204318	11,91833	0,174325	0,142568	1,621813	0,117017	0,321556	18,34246	10,93927	3,829715	0,490999	0,215864	23,87832	1,440429	13,7440142	34,3567813	25,533772	26,3654325	20,8570704	5,50836218	12,8112656	96,954169
