# Supplementary material for: Physical activity and IgG N-glycosylation in medical students: a cross-sectional study
Source: Croat Med J. 2026 Jun;67(3):156–63. doi: 10.3325/cmj.2026.67.156 (PMC13247745; doi:10.3325/cmj.2026.67.156)
Supplement: Supplementary Table 3 [file CroatMedJ_67_s016.pdf]

Supplemental Table 3. Definition and structural composition of derived IgG glycan traits.

IgG N-glycan trait	Structural definition
G0 (agalactosylation)	Proportion of agalactosylated IgG glycan (P14+P15+P18)
G1 (monogalactosylation)	Proportion of monogalactosylated IgG glycan structures (P8+P16+P17+P19+P20+P21+P22+P23+P24)
G2 (digalactosylation)	Proportion of digalactosylated IgG glycan structures (P1+P2+P3+P4+P9+P10+P11+P12+P25+P26+P27)
S0 (asialylation)	Proportion of asialylated IgG glycan structures (P14+P15+P16+P17+P18+P19+P20+P21+P22+P23+P24+P25+P26+P27)
S1 (monosialylation)	Proportion of monosialylated IgG glycan structures (P5+P6+P7+P8+P9+P10+P11+P12+P13)
S2 (disialylation)	Proportion of disialylated IgG glycan structures (P1+P2+P3+P4)
B (bisecting GlcNAc)	Proportion of IgG glycan structures containing a bisecting N-acetylglucosamine (P2+P4+P11+P13+P14+P18+P19+P20+P23+P24+P25+P27)
CF (core-fucosylation)	Proportion of core-fucosylated IgG glycan structures (P3+P4+P7+P12+P13 +P15+P18+P21+P22+P23+P24+P26+P27)
