# Supplementary material for: Physical activity and IgG N-glycosylation in medical students: a cross-sectional study
Source: Croat Med J. 2026 Jun;67(3):156–63. doi: 10.3325/cmj.2026.67.156 (PMC13247745; doi:10.3325/cmj.2026.67.156)
Supplement: Supplementary Table 4 [file CroatMedJ_67_s017.pdf]

Supplemental Table 4. Associations between physical activity (PA) domains assessed by the IPAQ-SF and IgG N-glycans.

	Vigorous PA (MET-min/week)		Moderate PA (MET-min/week)		Walking (MET-min/week)		Total PA (MET-min/week)		Sitting time (minutes)	
	ρ	*P*	ρ	*P*	ρ	*P*	ρ	*P*	ρ	*P*
P1	-0.07	0.517	0.02	0.828	0.06	0.602	0.01	0.937	-0.07	0.564
P2	-0.07	0.532	0.13	0.269	0.04	0.757	0.04	0.751	0.04	0.718
P3	-0.03	0.793	0.12	0.272	0.08	0.507	0.03	0.766	0.00	0.979
P4	0.10	0.401	0.21	0.068	0.00	0.997	0.11	0.327	-0.07	0.550
P5	-0.15	0.174	0.14	0.226	0.00	0.989	-0.13	0.245	0.09	0.411
P6	-0.10	0.398	-0.01	0.911	0.02	0.827	-0.02	0.860	0.11	0.338
P7	0.11	0.330	0.15	0.189	0.13	0.243	0.24	**0.033**	0.13	0.255
P8	-0.03	0.784	0.04	0.749	0.35	**0.002**	0.15	0.177	0.03	0.791
P9	0.00	0.982	0.13	0.269	-0.04	0.711	0.07	0.519	0.03	0.815
P10	-0.08	0.504	-0.01	0.940	-0.05	0.666	-0.05	0.679	0.07	0.569
P11	-0.14	0.233	0.20	0.080	-0.10	0.385	-0.13	0.268	0.02	0.856
P12	-0.12	0.288	-0.07	0.522	-0.07	0.528	-0.18	0.120	0.20	0.081
P13	0.16	0.159	0.20	0.073	-0.03	0.818	0.13	0.265	-0.22	0.046
P14	0.04	0.714	0.22	0.051	0.02	0.877	0.18	0.117	-0.02	0.841
P15	0.09	0.419	0.09	0.430	0.17	0.131	0.22	0.056	-0.17	0.129
P16	-0.07	0.560	-0.01	0.962	-0.02	0.837	-0.02	0.844	0.08	0.506
P17	-0.03	0.806	0.02	0.838	0.01	0.952	0.01	0.938	0.07	0.563
P18	-0.03	0.809	-0.04	0.713	0.13	0.272	0.12	0.295	-0.12	0.304
P19	-0.07	0.533	0.07	0.539	-0.03	0.787	-0.01	0.956	0.03	0.811
P20	0.07	0.565	0.07	0.537	-0.06	0.587	0.07	0.536	0.05	0.689
P21	0.19	0.099	0.11	0.322	0.02	0.844	0.23	**0.041**	-0.08	0.464
P22	0.17	0.139	0.06	0.571	0.24	**0.037**	0.22	0.053	-0.16	0.151
P23	-0.03	0.789	-0.04	0.758	-0.21	0.061	-0.12	0.282	0.09	0.422
P24	0.04	0.753	0.03	0.785	0.06	0.589	0.09	0.413	-0.10	0.360
P25	0.04	0.718	0.25	**0.024**	-0.18	0.120	-0.08	0.496	0.02	0.844
P26	-0.15	0.196	-0.22	0.056	-0.21	0.059	-0.33	**0.003**	0.14	0.208
P27	-0.03	0.795	-0.01	0.915	-0.26	**0.020**	-0.21	0.068	0.05	0.693

MET-min/week = metabolic equivalent minutes per week; ρ = Spearman's rank correlation coefficients; *P* = *P*-value
