# Supplementary material for: Physical activity and IgG N-glycosylation in medical students: a cross-sectional study
Source: Croat Med J. 2026 Jun;67(3):156–63. doi: 10.3325/cmj.2026.67.156 (PMC13247745; doi:10.3325/cmj.2026.67.156)
Supplement: Supplementary Table 5 [file CroatMedJ_67_s018.pdf]

Supplemental Table 5. Associations between physical activity (IPAQ-SF) and derived IgG N-glycan traits: unadjusted and BMI-adjusted correlations

	Vigorous PA (MET-min/week)		Moderate PA (MET-min/week)		Walking (MET-min/week)		Total PA (MET-min/week)		Sitting time (minutes)	
	ρ	*P*	ρ	*P*	ρ	*P*	ρ	*P*	ρ	*P*
G0	0.06	0.620	0.06	0.623	0.19	0.102	0.19	0.085	-0.17	0.127
G0*	0.072	0.527	−0.001	0.994	0.164	0.150	0.175	0.123	−0.156	0.170
G1	0.24	**0.034**	0.14	0.222	0.05	0.640	0.27	**0.017**	-0.10	0.382
G1*	0.242	**0.032**	0.107	0.347	0.041	0.723	0.254	**0.024**	−0.096	0.398
G2	-0.15	0.190	-0.21	0.062	-0.22	0.051	-0.34	**0.002**	0.15	0.189
G2*	−0.140	0.218	−0.144	0.206	−0.225	**0.046**	−0.310	**0.005**	0.137	0.227
S0	-0.08	0.472	0.04	0.720	-0.01	0.955	-0.07	0.518	0.15	0.182
S0*	−0.088	0.441	0.071	0.532	−0.011	0.921	−0.069	0.544	0.139	0.221
S1	-0.09	0.423	-0.00	0.994	-0.03	0.781	-0.12	0.312	0.16	0.150
S1*	−0.112	0.327	0.035	0.761	−0.022	0.849	−0.107	0.346	0.165	0.146
S2	0.00	0.988	0.17	0.125	0.06	0.615	0.07	0.557	-0.01	0.899
S2*	−0.015	0.897	0.158	0.164	0.068	0.550	0.059	0.605	−0.015	0.893
B	0.02	0.859	0.10	0.358	-0.15	0.192	0.00	0.984	-0.03	0.773
B*	0.008	0.941	0.074	0.514	−0.154	0.175	−0.016	0.887	−0.046	0.685
CF	0.09	0.447	-0.13	0.257	0.01	0.907	-0.03	0.824	-0.06	0.624
CF*	0.085	0.457	−0.124	0.278	0.009	0.940	−0.025	0.824	−0.059	0.608

MET-min/week = metabolic equivalent minutes per week; *P* = P-value; ρ = Spearman's rank correlation coefficients
*Partial Spearman correlations were adjusted for body mass index using residualisation
